# Adverse events from spinal manipulation in the pregnant and postpartum periods: a critical review of the literature

**DOI:** 10.1186/2045-709X-20-8

**Published:** 2012-03-28

**Authors:** Kent Jason Stuber, Shari Wynd, Carol Ann Weis

**Affiliations:** 1 Division of Graduate Education and Research, Canadian Memorial Chiropractic College, 6100 Leslie Street, Toronto, ON M2H 3J1, Canada; 2 Texas Chiropractic College, 5912 Spencer Highway, Pasadena, TX 77505-1699, USA

**Keywords:** Chiropractic, Spinal manipulative Therapy, Pregnancy, Postpartum, Adverse effects

## Abstract

**Background:**

The safety of spinal manipulation during pregnancy and the postpartum periods has been a matter of debate among manual therapists. Spinal manipulative therapy during these periods is a commonly performed intervention as musculoskeletal pain is common in these patients. To date there has not been an evaluation of the literature on this topic exclusively.

**Methods:**

A literature search was conducted on PubMed, CINAHL and the Index to Chiropractic Literature along with reference searching for articles published in English and French in the peer-reviewed literature that documented adverse effects of spinal manipulation during either pregnancy or postpartum. Case reports, case series, and any other clinical study designs were deemed acceptable for inclusion, as were systematic reviews. The appropriate Scottish Intercollegiate Guidelines Network (SIGN) tools were used to rate included articles for quality when applicable.

**Results:**

Five articles identifying adverse events in seven subjects following spinal manipulation were included in this review, along with two systematic reviews. The articles were published between 1978 and 2009. Two articles describing adverse effects from spinal manipulation on two postpartum patients were included, while the remaining three articles on five patients with adverse effects following spinal manipulation were on pregnant patients. Injury severity ranged from minor injury such as increasing pain after treatment that resolved within a few days to more severe injuries including fracture, stroke, and epidural hematoma. SIGN scores of the prospective observational cohort study and systematic reviews indicated acceptable quality.

**Conclusions:**

There are only a few reported cases of adverse events following spinal manipulation during pregnancy and the postpartum period identified in the literature. While improved reporting of such events is required in the future, it may be that such injuries are relatively rare.

## Background

Musculoskeletal pain is a common occurrence during pregnancy and the postpartum period. For example, low back pain has been reported to occur as frequently as 50% to 85% of pregnant women [1-4] and at two to three years postpartum, eight to 20% of these women still report persistent symptoms [5,6]. Although low back pain is often accepted as an unavoidable complaint during pregnancy, for some women the pain can be debilitating, interfering with sleep, work and normal activities of daily living [7,8]. However, the etiology of this pain is unknown [9]. It has been suggested that low back pain experienced during pregnancy is multifactoral in nature and some of the proposed mechanisms include, but are not limited to, the influence of altered circulating relaxin levels producing ligamentous laxity [7,10], maternal weight gain and/or biomechanical changes due to pregnancy [7].

In the non-pregnant population, low back pain is a significant cause of pain and disability as well, with 80% of the population experiencing an episode during their lifetime [7,11]. Neck pain [12,13] and headaches [14] are also a substantial source of pain and disability in the non-pregnant population [12]. One of the effective treatment options used by manual practitioners for those suffering from low back pain [15,16] cervical spine [16,17] and some headache pain [18,19] is spinal manipulative therapy (SMT). SMT is usually characterized as a localized force of high velocity and low amplitude directed at a spinal segment [1]. Severe adverse effects of SMT are rare in the cervical spine [20-22] and lumbar spine [23].

Manual treatment options for pregnancy-induced pain, such as back pain, have been reported to be limited [1]. However, chiropractors report seeing pregnant patients frequently, and surveys of chiropractors reflect an opinion that SMT is safe for pregnant patients [1,24]. While the safety of SMT for adult and pediatric populations has undergone scrutiny in both public and scientific domains [13,22,25], the safety of SMT in sub-groups of the population including pregnant and postpartum patients has received little attention. This lack of evidence is surprising given the obvious importance of the welfare of the expectant and new mother. Given the hormonal and the coagulability status of peripartum and postpartum individuals, it is possible that SMT is a contraindication to the musculoskeletal complaints associated with pregnancy.

It is accepted that females are more susceptible to increases in joint laxity than men [26-28]. Hormonal causes have been postulated as a potential source for this increase in female joint laxity [29-32]. Relaxin, a polypeptide that is produced by the corpus luteum during pregnancy [32], is one of the implicated hormones. In the pregnant female, relaxin is essential in order to secure the passage of the fetus during parturition in several animal species [33]; it has been associated with a decrease in soft tissue tension especially in preparing the female body for delivery including relaxing the pelvic ligaments, inhibiting spontaneous uterine contractions, ripening of the uterine cervix, and stimulating the mammary glands [34]. Although relaxin increases laxity in the symphysis pubis in preparation for birth, its effects are not solely limited to that joint. In addition, women immediately postpartum are thought to also have this hormone-mediated ligament laxity that might reduce the protective stability of the intervertebral articulations [35].

Hypercoaguable disorders that promote thrombosis have been categorized as thrombophilias [36]. During pregnancy and the postpartum state the risk of thrombophilia increases compared to the non-pregnant state [36,37]. Thromboembolism or pulmonary embolism has been identified as the leading cause of maternal death in the United States [36,37]. These hypercoaguable disorders during pregnancy can be a result of venous stasis, changes in the vessel wall and changes in the composition of blood; also known as Virchow's triad [36].

In the absence of a prospective study of the safety of SMT during the antepartum and postpartum periods, it would be beneficial to survey the scientific literature for the number and types of injuries sustained by pregnant and postpartum patients following spinal manipulation. While systematic reviews of the literature on the use of SMT for pregnancy and related conditions have been conducted [1,11], an exploration of the literature specifically for adverse events associated with SMT and pregnancy has not yet been undertaken. Accordingly, the aim of this study is to critically review the literature for reported cases of iatrogenic injuries following spinal manipulative therapy during the pregnancy and postpartum periods.

## Methods

### Objective

To collect and synthesize available evidence on adverse events associated with SMT during the pregnancy and postpartum periods.

### Search strategy

A literature search was conducted in three different electronic databases: PubMed (including MEDLINE), CINAHL, and the Index to Chiropractic Literature. The date ranges applied were from the beginning of each respective database to October 2011. The following search terms were employed: adverse effects, adverse reactions, adverse events, harm, pregnancy, postpartum, chiropractic, spinal manipulative therapy, spinal manipulation, and manual therapy. These search terms were categorized and combined using Boolean terms (please see Additional file 1: Appendix 1 for the complete search strategy). Reference searching of relevant articles retrieved from the electronic literature search was also undertaken, as was a search of each of the authors' personal collections. Specific inclusion criteria for this review were:

#### Study designs

All clinical study designs published in peer-reviewed journals. Conference proceedings, cross-sectional and other descriptive designs and narrative reviews were excluded.

#### Population

Female patients who are either pregnant or postpartum (defined as the period between the delivery of the child and six weeks after the birth) [38].

#### Intervention

Spinal Manipulative Therapy (SMT) (defined as a manual therapy technique that uses a high velocity low amplitude thrust applied at a spinal motion segment [39] to any region of the spine.

#### Comparison

Not relevant.

#### Outcomes

Any adverse events associated with SMT.

#### Language

Articles in either English or French were considered for inclusion.

### Study selection

Two of the authors (KS and SW) independently reviewed the titles and abstracts of the electronic database searches for any that appeared to match the inclusion criteria. The full text versions of any potentially relevant articles were obtained and reviewed by the same two authors using the inclusion criteria described above. Each of the authors compiled a list of articles to include that was compared, and any disagreements were resolved through discussion.

### Data collection and rating process

A data extraction sheet was compiled by one of the authors (KS) and relevant data from each included article was entered into the sheet. The appropriate Scottish Intercollegiate Guidelines Network (SIGN) tools were used to rate included articles for quality, although any case reports included in the review were not rated for quality as there is no applicable SIGN tool for case reports. The overall assessment of a paper using a SIGN tool is given one of three scores: "++" indicates the highest level of methodological quality for that study type, fulfilling all or most of the internal validity criteria for that particular study type, "+" indicates some criteria were fulfilled, and "-" indicates that few or none of the criteria were satisfied. Two of the authors (KS and SW) rated the articles using the applicable SIGN tool. One of the authors (KS) was an author on one of the systematic reviews and thus that article was rated by the other two authors (SW and CAW) to avoid potential bias. Where reviewers disagreed, consensus was achieved by discussion.

### Analysis

Meta-analysis could not be conducted as only case reports and an observational cohort study were identified; data are summarized in text (percentages).

## Results

**Figure 1:**
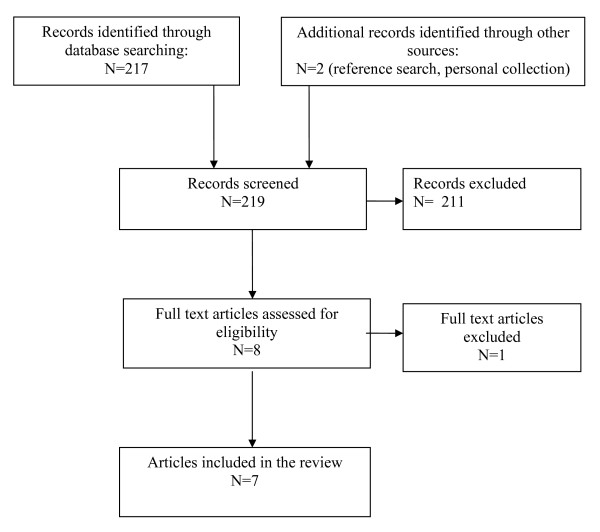
**PRISMA Information Flow **[40].

**Figure 2:**
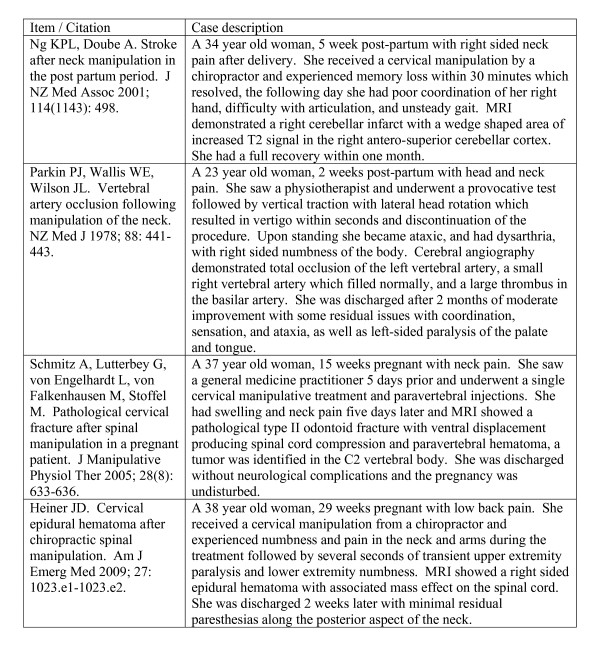
**Case Report Descriptions**.

Figure 1 depicts the flow of articles through the review process [40]. One full text article was excluded because it did not use HVLA spinal manipulation [41]. Five articles that identified possible adverse events in seven subjects following spinal manipulation of a pregnant or post-partum subject were identified from the literature search, along with two relevant systematic reviews [1,11] (the full list of included articles appears in Additional file 2: Appendix 2). The five articles consisted of four case reports [20,21,35,42] and one prospective observational cohort study [43]. The four case reports all detailed adverse events following cervical manipulation, whereas the prospective observational cohort study described three adverse events following lumbar manipulation. The articles were published between 1978 and 2009. Figure 2 describes the four case reports included, while (Additional file 3: Table S1) provides additional details on those case reports. One case report [42] was identified by reference searching. A systematic review of manipulative therapy during pregnancy [1] was identified from the authors' personal collections.

Murphy et al published an observational cohort study in 2009, which evaluated a diagnosis-based decision rule for pregnancy-related lumbopelvic pain [43]. One hundred and fifteen patients began the study and complete data was obtained on 78 subjects. In terms of adverse effects three subjects (3.8%) reported increased pain after treatment; this was reported as resolving for two of these patients in less than 48 hours and in one week for the third patient. The treatments that each patient received depended on their specific diagnosis and it is unsure what diagnosis these patients were given. However nearly all patients (68/78) received some form of manual therapy. No other complications were reported in this study. The overall SIGN rating assigned to this article was "+", indicating acceptable quality although it was limited by a lack of blinding and possible confounders.

The systematic review by Stuber and Smith in 2008 on spinal manipulation for pregnancy-related lower back pain indicated that no adverse effects were noted in any of the papers that they reviewed, although only one of the articles specifically commented on an absence of adverse events [11]. The systematic review by Khorsan et al published in 2009 identified only the case report by Schmitz et al as having an adverse event resulting from manipulative therapy during pregnancy [1]. Most of the studies included in that review did not report adverse effects at all, although it was noted that three clinical studies indicated that there were no adverse events during their trials. The overall SIGN rating assigned to both of these systematic reviews was "++", indicating good quality.

## Discussion

To our knowledge, this is the first critical review of the literature regarding adverse events from spinal manipulation during pregnancy and postpartum and it provides healthcare professionals with a comprehensive evaluation of the available scientific literature. This review revealed adverse affects during spinal manipulation in three studies during pregnancy and two studies in the postpartum period. Of the studies identified, four case studies demonstrated adverse events following cervical manipulation whereas the observational cohort study demonstrated adverse events following lumbar manipulation. The remaining two papers that were identified were systematic reviews. As such reports of adverse events following spinal manipulation in these populations are scarce in the literature.

Mild and transient adverse events were reported as a result of lumbar spinal manipulation [43] whereas the serious adverse events reported in the literature all occurred following cervical spinal manipulation either during pregnancy [20,21] or postpartum [35,42]. Murphy and colleagues found that 3.4% of their pregnant population incurred an injury following manual therapy which resulted in a transient increase in pain after a single session. They found this to be much lower than other studies that have focused on manual therapy in which the rate of injury was approximately one third of the study group [43]. As such they suggested that SMT of the lumbar spine is safe for this population, however a larger sample size would be needed to detect rare complications [43]. This review did not identify any injuries or adverse events that could be associated specifically with either being pregnant or in the postpartum period (such as premature delivery or an abnormally difficult delivery, etc), that is to say that the adverse events identified may have occurred in a non-pregnant patient.

In the non-pregnant population severe complications after cervical spinal manipulation are rare [13,22]. However rare, reported complications include vertebral artery dissection, cord or root injury, epidural hematoma, cervical disc rupture and vertebral fracture [21]. Pregnant and postpartum populations are not immune to this possibility and given the hormonal and coagulability status of pregnant and postpartum patients it is possible that SMT is contraindicated in pregnant and postpartum patients with musculoskeletal complaints. However, this may depend on the spinal regions and complaints being treated as different consideration may need to be given to manipulation of the cervical spine versus the thoracic or lumbopelvic regions.

Most contraindications to spinal manipulation are evident during a careful history and physical exam [21]. Clinicians who use SMT as part of the plan of management for pregnant patients should consider prothrombotic and joint laxity risk factors when deciding whether to undertake such a therapy in order to minimize the risks of potentially dangerous neurological complications [35]. Patients at higher risk for complications, such as those in a post-thrombotic state and possibly those with lax joints, should be treated with additional care and consideration. There is an increased importance to counsel this patient with respect to the risks of SMT [20,35] and these patients should be made aware of the signs and symptoms of possible neurovascular complications [20,35]. However, based on the literature reviewed, it cannot be ascertained as to what role those factors may have actually played in the etiology of the adverse events documented, if any.

Although this study has resulted in very few papers to review, it had strengths including the thorough search of the literature to help reduce bias in the review. The authors searched multiple relevant electronic databases over all possible years represented in those databases, employed a number of broad search terms, performed reference and hand-searching, examined personal libraries, and used multiple authors to determine articles for inclusion in the review and to evaluate and rate the literature.

The major limitation of this critical review was the number of studies available and the hierarchy of evidence that the studies available yielded. The papers identified for this review were case studies and a prospective observational cohort study, both of which are lower levels of evidence. Given the levels and paucity of evidence identified, the possible level of risk to pregnant and postpartum patients undergoing spinal manipulative therapy cannot be measured or stated definitively, nor can it be determined if any such risk level is higher or lower than in the non-pregnant or postpartum populations. However, this does allow for hypothesis generation and should help drive future directions for research. There is a need to design and execute larger and higher quality observational and randomized controlled studies investigating the potential benefits of the use of spinal manipulation as a treatment during pregnancy or postpartum particularly for those with low back pain. Such studies should ensure that any possible adverse events are tracked throughout. One possible option for an observational study design may be a case-crossover study as adverse events from spinal manipulative therapy may be rare in these populations [44,45]. The previous systematic reviews on spinal manipulation as a treatment during pregnancy have highlighted the lower levels of evidence available thus far on the topic [1,11]. Another limitation of this review was including only articles published in English and French.

## Conclusions

There are only a handful of reported cases of adverse events following spinal manipulation during pregnancy and the postpartum period in the literature with the severity ranging from mild increases in pain that resolved quickly to significant life-threatening injuries. While improved reporting of such events is required in the future, it may be that such injuries are relatively rare. Clearly future research into efficacy of this treatment for these populations and the rates of occurrence of adverse events is necessary to determine whether or not this is true.

## Competing interests

The authors declare that they have no competing interests.

## Authors' contributions

KJS conceived the study, participated in its design and coordination, conducted the literature search, screened the literature search results, analyzed and interpreted the data, and drafted the manuscript. SW conceived the study, participated in its design, screened the literature search results, and analyzed and interpreted the data. CAW analyzed and interpreted the data and drafted the manuscript. All authors read and approved the final manuscript.

## Supplementary Material

Additional file 1 **Appendix 1**. Search strategy. Click here for file

Additional file 2 **Appendix 2**. List of included articles. Click here for file

Additional file 3 **Table S1**. Case report details. Click here for file

## References

[B1] Khorsan R Hawk C Lisi AJ Kizhakkeveettil A Manipulative therapy for pregnancy and related conditions: a systematic review Obstet Gynecol Survey 2009 64 416 427 10.1097/OGX.0b013e31819f9ddf 19445815

[B2] Skaggs C Prather H Gross G George J Thompson P Nelson D Back and pelvis pain in an underserved United States pregnant population: A preliminary descriptive survey J Manipulative Physiol Ther 2007 30 130 134 10.1016/j.jmpt.2006.12.008 17320734

[B3] Stapleton D MacLennan A Kristiansson P The prevalence of recalled low back pain during and after pregnancy: a South Australian population survey Aust N Z J Obstet Gynaecol 2002 42 482 485 10.1111/j.0004-8666.2002.00482.x 12495090

[B4] Wu H Meijer O Uegaki K Mens J van Dieen J Wuisman P Ostgaard H Pregnancy-related pelvic girdle pain (PPP) I: Terminology, clinical presentation and prevalence Eur Spine J 2004 13 575 589 10.1007/s00586-003-0615-y 15338362 PMC3476662

[B5] Olsen MF Gutke A Elden H Nordenman C Fabrisius L Gravesen M Lind A Kjellby-Wendt G Self-administered tests as a screening procedure for pregnancy-related pelvic girdle pain Eur Spine J 2009 18 1121 1129 10.1007/s00586-009-0948-2 19330361 PMC2899498

[B6] Mogren IM Physical activity and persistent low back pain and pelvic pain post partum BMC Public Health 2008 8 417 421 10.1186/1471-2458-8-417 19102737 PMC2630950

[B7] Pennick VE Young G Interventions for preventing and treating pelvic and back pain in pregnancy Cochrane Database of Syst Rev 2007 2 CD001139 DOI:10.1002/14651858.CD001139.pub2 17443503 10.1002/14651858.CD001139.pub2

[B8] Mogren IM Pohjanen AI Low back pain and pelvic pain during pregnancy: Prevalence and risk factors Spine 2005 30 983 991 10.1097/01.brs.0000158957.42198.8e 15834344

[B9] Lisi A Chiropractic spinal manipulation for low back pain of pregnancy; A retrospective case series J Midwifery Women Health 2005 51 e7 e10 10.1016/j.jmwh.2005.09.001 16399602

[B10] Kristiansson P Savardsudd K von Schoultz B Back pain during pregnancy A prospective study. Spine 1996 2 702 709 10.1097/00007632-199603150-00008 8882692

[B11] Stuber KJ Smith DL Chiropractic treatment of pregnancy-related low back pain: a systematic review of the evidence J Manipulative Physiol Ther 2008 31 447 454 10.1016/j.jmpt.2008.06.009 18722200

[B12] Natvig B Ihlebaek C Grotle M Brage S Bruugsgaard D Neck pain is often a part of widespread pain and is associated with reduced functioning Spine 2010 35 E1285 E1289 20938391 10.1097/BRS.0b013e3181e38e73

[B13] Haldeman S Carroll L Cassidy JD Findings from The Bone and Joint Decade 2000 to 2010 Task Force on Neck Pain and Its Associated Disorders J Occup Environ Med 2010 52 424 427 10.1097/JOM.0b013e3181d44f3b 20357682

[B14] Coulter ID Hurwitz EL Adams AH Patients using chiropractors in North America: who are they, and why are they in chiropractic care? Spine 2002 27 291 296 10.1097/00007632-200202010-00018 11805694

[B15] Bishop PB Quon JA Fisher CG Dvorak MF The Chiropractic Hospital-based Interventions Research Outcomes (CHIRO) study: a randomized controlled trial on the effectiveness of clinical practice guidelines in the medical and chiropractic management of patients with acute mechanical low back pain Spine J 2010 10 1055 64 10.1016/j.spinee.2010.08.019 20889389

[B16] McMorland G Suter E Chiropractic management of mechanical neck and low -back pain: a retrospective, outcome-based analysis J Manipulative Physiol Ther 2000 23 307 11 10863249

[B17] Shekelle PG Coulter I Cervical spine manipulatiion: summary report of a systemic review of the literature and multidisciplinary expert panel J Spin Disord 1997 10 223 8 9213278

[B18] Nelson C Principles of effective headache management Topics Clin Chiro 1998 5 55 61

[B19] Bryans R Descarreaux M Duranleau M Marcoux H Potter B Ruegg R Shaw L Watkin R White E Evidence-based guidelines for the chiropractic treatment of adults with headache J Manipulative Physiol Ther 2011 34 274 89 10.1016/j.jmpt.2011.04.008 21640251

[B20] Heiner JD Cervical epidural hematoma after chiropractic spinal manipulation Am J Emerg Med 2009 27 1023. e1 1023.e2 19857440 10.1016/j.ajem.2008.12.031

[B21] Schmitz A Lutterbey G von Engelhardt L von Falkenhausen M Stoffel M Pathological cervical fracture after spinal manipulation in a pregnant patient J Manipulative Physiol Ther 2005 28 633 636 10.1016/j.jmpt.2005.08.017 16226634

[B22] Cassidy JD Boyle E Côté P He Y Hogg-Johnson S Silver FL Bondy SJ Risk of vertebrobasilar stroke and chiropractic care: results of a population-based case-control and case-crossover study J Manipulative Physiol Ther 2009 32 Suppl 2 S201 8 19251066 10.1016/j.jmpt.2008.11.020

[B23] Oliphant D Safety of spinal manipulation in the treatment of lumbar disc herniations: a systematic review and risk assessment J Manipulative Physiol Ther 2004 27 197 210 10.1016/j.jmpt.2003.12.023 15129202

[B24] Stuber K The safety of chiropractic during pregnancy: a pilot e-mail survey of chiropractors' opinions Clin Chiro 2007 10 24 35 10.1016/j.clch.2006.10.003

[B25] Humphreys BK Possible adverse events in children treated by manual therapy: a review Chiropr Osteopat 2010 18 12 20525194 10.1186/1746-1340-18-12 PMC2890687

[B26] Juul-Kristensen B Rogind H Jensen DV Remvig L Inter-examiner reproducibility of tests and criteria for generalized hypermobility and benign joint hypermobility syndrome Rheumatol 2007 46 1835 1841 10.1093/rheumatology/kem290 18006569

[B27] Beighton P Solomon L Soskolne CL Articular mobility in an African population Ann Rheum Dis 1973 32 413 418 10.1136/ard.32.5.413 4751776 PMC1006136

[B28] Carter C Wilkinson J Persistent joint laxity and congenital dislocation J Bone Joint Surg 1964 46B 40 45 14126235

[B29] Marnach ML Ramin KD Ramsey PS Song SW Stensland JJ An KN Characterization of the relationship between joint laxity and maternal hormones in pregnancy Am Coll Obstet Gynecol 2003 101 331 335 10.1016/s0029-7844(02)02447-x 12576258

[B30] Björklund K Nordström ML Odlind V Combined oral contraceptives do not increase the risk of back and pelvic pain during pregnancy or after delivery Acta Obstet Gynecol Scand 2000 79 979 83 11081684

[B31] Calguneri M Bird HA Wright AV Changes in joint laxity occurring during pregnancy Ann Rheum Dis 1982 41 126 128 10.1136/ard.41.2.126 7073339 PMC1000894

[B32] Dragoo JL Lee RS Benhaim P Finerman GA Hame SL Relaxin receptors in the human female anterior cruciate ligament Am J Sports Med 2003 31 577 84 12860548 10.1177/03635465030310041701

[B33] Hansen A Jensen DV Larsen E Wilken-Jensen C Petersen K Relaxin is not related to symptom-giving pelvic relaxation in pregnant women Acta Obstet Gynecol Scand 1996 75 245 249 10.3109/00016349609047095 8607337

[B34] Skott O Carter AM Relaxin is a vasodilator hormone Am J Physiol Regularory Integrative Comp Care 2002 283 R347 R348 10.1152/ajpregu.00264.2002 12121846

[B35] Ng KPL Doube A Stroke after neck manipulation in the post partum period J NZ Med Assoc 2001 114 498 11797875

[B36] Stella CL Sibai BM Thrombophilia and adverse maternal-perinatal outcome Clin Obstet Gynecol 2006 49 850 860 10.1097/01.grf.0000211954.66959.e1 17082680

[B37] Drife J Thromboembolism. Br Med Bull 2003 67 177 190 10.1093/bmb/ldg010 14711763

[B38] Maternal and Newborn Health/Safe Motherhood Unit, World Health Organization Postpartum Care of the Mother and Newborn: A Practical Guide. Geneva 1998

[B39] Bergmann T Peterson D Chiropractic Technique: Principles and Procedures 2011 3 St. Louis: Elsevier

[B40] Moher D Liberati A Tetzlaff J Altman DG Preferred reporting items for systematic reviews 368 and meta-analyses: the PRISMA statement J Clin Epidemiol 2009 62 1006 1012 10.1016/j.jclinepi.2009.06.005 19631508

[B41] McPartland JM Craniosacral iatriogenesis - side-effects from cranial sacral treatment: case reports and commentary J Bodywork Movement Ther 1996 1 2 5 10.1016/S1360-8592(96)80003-9

[B42] Parkin PJ Wallis WE Wilson JL Vertebral artery occlusion following manipulation of the neck NZ Med J 1978 88 441 443 281639

[B43] Murphy DR Hurwitz EL McGovern EE Outcome of pregnancy-related lumbopelvic pain treated according to a diagnosis-based decision rule: a prospective observational cohort study J Manipulative Physiol Ther 2009 32 616 624 10.1016/j.jmpt.2009.09.002 19836597

[B44] Redelmeier DA Tibshirani RJ Interpretation and bias in case-crossover studies J Clin Epidemiol 1997 50 1281 7 10.1016/S0895-4356(97)00196-0 9393384

[B45] Smeeth L Donnan PT Cook DG The use of primary care databases: case-control and case-only designs Fam Pract 2006 23 597 604 10.1093/fampra/cml025 16787956

