# Supplementary material for: Adverse events from spinal manipulation in the pregnant and postpartum periods: a critical review of the literature
Source: Chiropr Man Therap. 2012 Mar 28;20:8. doi: 10.1186/2045-709X-20-8 (PMC3348005; doi:10.1186/2045-709X-20-8)
Supplement: Additional file 1 — Appendix 1. Search strategy. [file 2045-709X-20-8-S1.DOCX]

**APPENDIX 1. SEARCH STRATEGY**

1. Adverse events

2. Adverse effects

3. Harm

4. Adverse reaction

5. 1 OR 2 OR 3 OR 4

6. pregnancy

7. prenatal

8. post-partum

9. 6 OR 7 OR 8

10. chiropractic

11. spinal manipulation

12. spinal manipulative therapy

13. manual therapy

14. 10 OR 11 OR 12 OR 13

15. 5 AND 9 AND 14
