# Supplementary material for: Adverse events from spinal manipulation in the pregnant and postpartum periods: a critical review of the literature
Source: Chiropr Man Therap. 2012 Mar 28;20:8. doi: 10.1186/2045-709X-20-8 (PMC3348005; doi:10.1186/2045-709X-20-8)
Supplement: Additional file 2 — Appendix 2. List of included articles. [file 2045-709X-20-8-S2.DOCX]

**APPENDIX 2. LIST OF INCLUDED ARTICLES**

**CASE REPORTS**

Ng KPL, Doube A: **Stroke after neck manipulation in the post partum period.** *J NZ Med Assoc* 2001, **114**: 498.

Parkin PJ, Wallis WE, Wilson JL: **Vertebral artery occlusion following manipulation of the neck.** *NZ Med J* 1978, **88**: 441-443.

Schmitz A, Lutterbey G, von Engelhardt L, von Falkenhausen M, Stoffel M: **Pathological cervical fracture after spinal manipulation in a pregnant patient.**  *J Manipulative Physiol Ther* 2005, **28**: 633-636.

Heiner JD: **Cervical epidural hematoma after chiropractic spinal manipulation.** *Am J Emerg Med* 2009, **27**: 1023.e1-1023.e2.

**PROSPECTIVE OBSERVATIONAL COHORT STUDY**

Murphy DR, Hurwitz EL, McGovern EE: **Outcome of pregnancy-related lumbopelvic pain treated according to a diagnosis-based decision rule: a prospective observational cohort study.** *J Manipulative Physiol Ther* 2009, **32**: 616-624.

**SYSTEMATIC REVIEWS**

Khorsan R, Hawk C, Lisi AJ, Kizhakkeveettil A: **Manipulative therapy for pregnancy and related conditions: a systematic review.** *Obstet Gynecol Survey* 2009, **64**: 416-427.

Stuber KJ, Smith DL: **Chiropractic treatment of pregnancy-related low back pain: a systematic review of the evidence.** *J Manipulative Physiol Ther* 2008, **31**: 447-454.
