# Supplementary material for: Adverse events from spinal manipulation in the pregnant and postpartum periods: a critical review of the literature
Source: Chiropr Man Therap. 2012 Mar 28;20:8. doi: 10.1186/2045-709X-20-8 (PMC3348005; doi:10.1186/2045-709X-20-8)
Supplement: Additional file 3 — Table S1. Case report details. [file 2045-709X-20-8-S3.DOCX]

**TABLE 1. Case report details**

| Item/Citation | Ng KPL, Doube A. Stroke after neck manipulation in the post partum period. J NZ Med Assoc 2001; 114(1143): 498. | Parkin PJ, Wallis WE, Wilson JL. Vertebral artery occlusion following manipulation of the neck. NZ Med J 1978; 88: 441-443. | Schmitz A, Lutterbey G, von Engelhardt L, von Falkenhausen M, Stoffel M. Pathological cervical fracture after spinal manipulation in a pregnant patient. J Manipulative Physiol Ther 2005; 28(8): 633-636. | Heiner JD. Cervical epidural hematoma after chiropractic spinal manipulation. Am J Emerg Med 2009; 27: 1023.e1-1023.e2. |
| --- | --- | --- | --- | --- |
| Patient demographics | 34 year old woman, 5 weeks post-partum, baby healthy, normal pregnancy, vaginal delivery | 23 year old woman, 2 weeks post-partum | 37 year old woman, 15 weeks pregnant | 38 year old woman, 29 weeks pregnant |
| Presence of co-morbidities | No antecedent neurological history or prior thrombotic events, infrequent tension headaches, family history unremarkable, stopped smoking 4 years prior. | Not indicated | No history of trauma or tumor, pregnancy had been unremarkable. Patient was found to have an aneurismal bone cyst | Indicated as otherwise healthy |
| Preceding symptoms | Right sided neck pain following delivery, aggravated by neck movements during breast-feeding | Head and neck pain | Diffuse neck pain | Only pregnancy related low back pain indicated |
| Duration of preceding symptoms | Approximately 5 weeks | Not indicated | 2 weeks | Not indicated |
| Description of the location of the injury | MRI showed right cerebellar infarct with a wedge shaped areas of increased T2 signal in the right antero-superior cerebellar cortex. MRA was unremarkable. | Cerebral angiography performed 24 hours after the onset of symptoms showed total occlusion of the left vertebral artery high in the neck, as well as a small right vertebral artery which filled normally, and a well filled basilar artery containing a large thrombus in the lower portion extending almost as far as the origin of the anterio inferior cerebellar artery. | MRI showed pathological type II odontoid fracture with ventral displacement of the odontoid process leading to spinal cord compression. Paravertebral hematoma from C2-C4. A tumor was identified in the vertebral body of the axis. | MRI showed right sided epidural hematoma from the foramen magnum to C4 level with associated mass effect on the spinal cord |
| Description of treatment including technique | Cervical manipulation | Provocative test performed initially (head rotated to both sides and held at the extremes of movement for several seconds) followed by vertical traction combined with lateral head rotation. Vertigo developed within seconds and the manipulation was stopped | Single manipulative treatment of the cervical spine 5 days prior to presentation. Also received paravertebral injections from the same practitioner | Cervical manipulation |
| Treating clinician (profession, experience) | Chiropractor | Physiotherapist | General medical practitioner | Chiropractor |
| Number of manipulations prior to the event | Not indicated | Not indicated | First time | First time in the cervical spine, 2 times previously for pregnancy related low back pain. |
| Time to onset of symptoms after manipulation | 30 minutes – memory loss, this resolved after 90 minutes, the next day she had poor coordination of her right hand, difficulty with articulation, unsteady gait | Vertigo developed within seconds during the manipulation, which was stopped. Upon standing became ataxic, dysarthria, with right sided numbness of the body. | Presented with neck pain and paravertebral swelling 5 days after the manuever. | Short term onset of numbness and pain in the neck and arms during treatment, had several seconds of transient upper extremity paralysis and lower extremity numbness at onset |
| Final outcome | Full neurological recovery within one month | Discharged after 2 months with moderate improvement in conjugate gaze palsies, virtually complete recovery of left hemiparesis, modest improvement in coordination and ataxia. Left sided paralysis of the palpate and tongue and sensory disturbances were unchanged. | Patient was discharged with her pregnancy undisturbed and without neurological complications | 2 weeks after discharged symptoms had nearly resolved with minimal residual paresthesias along the posterior aspect of the neck. |
